# Environmental distribution and genetic diversity of vegetative compatibility groups determine biocontrol strategies to mitigate aflatoxin contamination of maize by *A* *spergillus flavus*

**DOI:** 10.1111/1751-7915.12324

**Published:** 2015-10-27

**Authors:** Joseph Atehnkeng, Matthias Donner, Peter S. Ojiambo, Babatunde Ikotun, Joao Augusto, Peter J. Cotty, Ranajit Bandyopadhyay

**Affiliations:** ^1^ Plant Pathology UnitInternational Institute of Tropical AgriculturePMB 5320IbadanNigeria; ^2^ Institute for Plant Diseases, Phytopathology and Nematology in Soil EcosystemsUniversity of BonnBonnGermany; ^3^ Department of Plant PathologyNorth Carolina State UniversityRaleighNorth CarolinaUSA; ^4^ Department of Crop Protection and Environmental BiologyUniversity of IbadanIbadanNigeria; ^5^ USDA‐ARSDivision of Plant Pathology and MicrobiologyDepartment of Plant SciencesUniversity of ArizonaTucsonArizonaUSA

## Abstract

Maize infected by aflatoxin‐producing *A* *spergillus flavus* may become contaminated with aflatoxins, and as a result, threaten human health, food security and farmers' income in developing countries where maize is a staple. Environmental distribution and genetic diversity of *A* *. flavus* can influence the effectiveness of atoxigenic isolates in mitigating aflatoxin contamination. However, such information has not been used to facilitate selection and deployment of atoxigenic isolates. A total of 35 isolates of *A* *. flavus* isolated from maize samples collected from three agro‐ecological zones of Nigeria were used in this study. Ecophysiological characteristics, distribution and genetic diversity of the isolates were determined to identify vegetative compatibility groups (VCGs). The generated data were used to inform selection and deployment of native atoxigenic isolates to mitigate aflatoxin contamination in maize. In co‐inoculation with toxigenic isolates, atoxigenic isolates reduced aflatoxin contamination in grain by > 96%. A total of 25 VCGs were inferred from the collected isolates based on complementation tests involving nitrate non‐utilizing (*nit^−^*) mutants. To determine genetic diversity and distribution of VCGs across agro‐ecological zones, 832 *nit^−^* mutants from 52 locations in 11 administrative districts were paired with one self‐complementary nitrate auxotroph tester‐pair for each VCG. Atoxigenic VCGs accounted for 81.1% of the 153 positive complementations recorded. Genetic diversity of VCGs was highest in the derived savannah agro‐ecological zone (*H* = 2.61) compared with the southern Guinea savannah (*H* = 1.90) and northern Guinea savannah (*H* = 0.94) zones. Genetic richness (*H* = 2.60) and evenness (*E*_5_ = 0.96) of VCGs were high across all agro‐ecological zones. Ten VCGs (40%) had members restricted to the original location of isolation, whereas 15 VCGs (60%) had members located between the original source of isolation and a distance > 400 km away. The present study identified widely distributed VCGs in Nigeria such as AV0222, AV3279, AV3304 and AV16127, whose atoxigenic members can be deployed for a region‐wide biocontrol of toxigenic isolates to reduce aflatoxin contamination in maize.

## Introduction

Maize is an important staple food in most countries in Africa including West Africa (Shiferaw *et al*., 2011) where infants being weaned off mothers' milk rely mostly on maize flour for nutrition. Maize is often invaded by *Aspergillus* fungal species before and after harvest and in storage. *Aspergillus* species are commonly found in the soil, which acts as source of primary inoculum for infecting developing maize kernels during the growing season (Horn, 2007). *Aspergillus flavus*, in particular, is distributed globally with a high frequency of occurrence in warm climates (Cotty *et al*., 1994), which favour the growth of the fungus. The fungus produces aflatoxin, a mycotoxin that is a potent carcinogen. Aflatoxin is also toxic to some domesticated animals and has been implicated in human aflatoxicosis (Peraica *et al*., 1999).

Isolates of *A. flavus* with different aflatoxin‐producing ability can interact in maize kernels to influence the rate of aflatoxin production by toxigenic isolates (Mehl and Cotty, 2011). In addition, *Aspergillus* communities inhabiting different substrates, fields and regions can vary widely in their ability to produce aflatoxin. Aflatoxin‐nonproducing genotypes, known as atoxigenic isolates, are common within *A. flavus* communities (Cotty and Sobek, 1997). Knowledge of these subpopulations in each region can be useful in identifying local management practices to reduce aflatoxin contamination. Biologically, *A. flavus* isolates produce only B‐aflatoxins and can be divided into two morphotypes; S‐strain, which forms numerous small sclerotia that average < 400 μm in diameter and produce high levels of aflatoxin, and the L‐strain, which forms fewer but large sclerotia measuring > 400 μm in diameter, and, on average, produce less aflatoxin (Bayman and Cotty, 1991a, 1991b). Most of the atoxigenic isolates of *A. flavus* belong to the latter group. Depending on the geographic origin, some isolates with S‐strain morphotype produce both B‐aflatoxins and G‐aflatoxins, whereas others produce only B‐aflatoxins (Cotty and Cardwell, 1999). Although the B‐ and G‐aflatoxin producers with S‐strain morphotype have been placed in different species (Varga *et al*., 2011), most isolates from West Africa with this chemotype have been classified to the S_BG_ taxon (Probst *et al*., 2014).

Exposure of humans and domesticated animals to aflatoxins is limited in the US and the EU, where regulations limiting the amount of aflatoxin in foods and feeds have been established (van Egmond *et al*., 2007) and are enforced. However, in developing countries, especially in Africa, regulations of aflatoxin in food and feed are either nonexistent or not enforced because much of the agricultural produce never enter official commercialization channels, but moves through local markets (Bandyopadhyay *et al*., 2007). This lack of regulation often results in severe aflatoxin poisoning as evidenced with the outbreak of acute aflatoxicosis associated with aflatoxin contaminated maize in Kenya that resulted in several human fatalities (Lewis *et al*., 2005). In Nigeria, aflatoxin‐monitoring schemes are rare, although high levels of aflatoxin contamination in maize at harvest and after storage have been reported (Udoh *et al*., 2000).

Toxigenic and atoxigenic isolates of *A. flavus* can be subdivided genetically by their vegetative incompatibility (Bayman and Cotty, 1991a). In plant pathogenic fungi, these subdivisions are often correlated with morphological features and pathogenicity (Puhalla, 1985). It has been suggested that recombination events of fungal isolates could lead to re‐assortment of the vegetative compatible alleles to yield new vegetative compatibility group (VCG) phenotypes (Leslie, 1996). Vegetative compatibility tests have clarified genetic relationships within many asexual fungal species including *Fusarium oxysporum* (Cai *et al*., 2003) and *Verticillium* species (Correll *et al*., 1988) and several *Aspergillus* species (Bayman and Cotty, 1991b). In *A. flavus*, VCGs are clonal lineages and no evidence for gene flow among VCGs has been reported in natural habitats (Grubisha and Cotty, 2010). Identification of differences in the toxigenicity and genetic diversity of *A. flavus* populations through VCGs may help in understanding the population dynamics and provide important information that could be used to improve the efficacy of biocontrol (Pildain *et al*., 2004). Further, a recent study using experimental crosses in the laboratory suggested that cryptic sexual reproduction can occur in *A. flavus* between individuals belonging to different VCGs, a process that can potentially generate diversity in aflatoxin chemotypes (Moore *et al*., 2013). Thus, information on VCG diversity ensures that biocontrol agents applied in the field will be genetically similar to local populations of *A. flavus* and thereby limit the potential for any cryptic sexual recombination, if it occurs in nature.

Application of biocontrol products in agricultural fields does not increase the overall quantities of *A. flavus* on the crop at harvest and there is a negative relationship between the incidence of the applied atoxigenic isolate and aflatoxin concentration (Cotty and Bayman, 1993). The most important mechanism for this type of biocontrol is the displacement of toxigenic isolates in the crop environment through founder effects and differential sporulation on substrates (Mehl *et al*., 2012). Crops are typically infected by multiple genotypes of *A. flavus* (e.g., Horn and Greene, 1995) and applied atoxigenic isolates may compete with toxigenic isolates during co‐infection and also interfere with aflatoxin contamination. For certain atoxigenic isolates, competitive exclusion is sufficient in explaining aflatoxin reduction during co‐infection (Hruska *et al*., 2014), a process that is aided by initial host contact (Mehl and Cotty, 2011). However, other atoxigenic isolates reduce aflatoxin significantly more during co‐infection than predicted by competitive exclusion alone. This occurs through unknown mechanisms and typically provides an additional 10–20% reduction in aflatoxin (Mehl and Cotty, 2010). Potential mechanisms for the latter include thigmo‐downregulation of aflatoxin biosynthesis (Huang *et al*., 2011) and differential ability among isolates to use nutrient resources (Mehl and Cotty, 2013). Wicklow and colleagues (2003) concluded that physical exclusion and/or competition for nutrients could be due to the inability of the competing isolates to form a cooperative mycelial network due to vegetative incompatibility. Subsequent work showed that the strength of VCG reactions between isolates belonging to the same VCG was negatively correlated with reduction in aflatoxin production (Wicklow and Horn, 2007). More research on a myriad of adaptive factors related to microbial dominance in ecosystems (Cray *et al*., 2013) is needed to enhance understanding of competitive exclusion in *Aspergillus* section *Flavi*.

The use of atoxigenic VCGs of *A. flavus* as biocontrol agents of toxigenic isolates to reduce aflatoxin contamination of agricultural commodities is a common practice in the US (Cotty *et al*., 2008; Dorner, 2008). However, systematic studies to improve the use of indigenous atoxigenic isolates of *A. flavus* in Africa to reduce aflatoxin contamination in maize are still lacking. Such studies are needed to facilitate practical use of biocontrol within the continent (Bandyopadhyay and Cardwell, 2003). Previously, several atoxigenic isolates of *A. flavus* were identified as potential biocontrol agents of toxigenic isolates to reduce aflatoxin contamination in maize in Nigeria (Atehnkeng *et al*., 2008a). We further characterized these atoxigenic isolates using molecular techniques (Donner *et al*., 2010) to provide information on the stability of the atoxigenic phenotype to facilitate the selection of potential biocontrol agents. Environmental distribution and genetic diversity of *A. flavus* can influence the effectiveness of atoxigenic isolates in mitigating aflatoxin contamination in maize. However, no studies have been conducted to determine the VCG diversity of the *A. flavus* populations in different agro‐ecological zones in Nigeria. Understanding the environmental distribution and genetic diversity of VCGs can greatly facilitate identification of potential biocontrol agents and their deployment at a local or regional scale (Mehl *et al*., 2012; Ehrlich, 2014). For example, atoxigenic VCGs that are widely distributed are likely to be effective in reducing aflatoxin contamination when deployed either locally or on a regional scale. Further, use of atoxigenic VCGs that are genetically similar to local soil populations of *Aspergillus* communities minimizes the potential for sexual recombination and can increase efficacy of biocontrol and result in sustainable biocontrol of toxigenic isolates (Lewis *et al*., 2013). Thus, the objective of the study was to determine the VCG diversity of selected isolates of *A. flavus* and establish their distribution across different locations and agro‐ecological zones in Nigeria to inform selection of atoxigenic isolates and deployment of biocontrol to reduce aflatoxin contamination in maize.

## Results

### Toxigenicity and aflatoxin profiles

Out of the 35 *A. flavus* isolates tested, 11 isolates produced aflatoxin and were classified as toxigenic, whereas 24 isolates did not produce any aflatoxin and were grouped as atoxigenic (Table 1). Atoxigenic isolates occurred in different locations in the three agro‐ecological zones, but a majority (83.3%) was found in the derived savannah (DS) zone (Table 1). Aflatoxin production in co‐inoculation experiments involving atoxigenic isolates and the highly toxigenic La3228 ranged from 17.1 to 499.3 ppb (Table 1). The corresponding reduction in aflatoxin in the co‐inoculation experiments was very high with the atoxigenic isolates reducing aflatoxin concentration in maize grain by > 96% (Table 1).

**Table 1:** Origin, profile of aflatoxin production and vegetative compatibility groups of *A* *spergillus flavus* isolates collected in Nigeria between 2008 and 2009^a^

Origin			Isolate designation		Aflatoxin B^c^		VCG
AEZ^b^	State	District	Code	Chemotype	Content (ppb)	RED (%)	
DS	FCT	Abuja	Ab2216	Toxigenic	–	–	AV2216
SGS	Niger	Bida	Bi1339	Toxigenic	–	–	AV2216
NGS	Kaduna	Saminaka	Ka16127	Atoxigenic	246.9	97.1	AV16127
SGS	Nassarawa	Akwanga	La2757	Atoxigenic	307.0	96.4	AV2757
SGS	Nassarawa	Akwanga	La3020	Toxigenic	–	–	AV3020
SGS	Nassarawa	Akwanga	La3058	Atoxigenic	109.4	98.7	AV3058
DS	Nassarawa	Lafia	La3108	Atoxigenic	153.0	98.2	AV3108
DS	Nassarawa	Lafia	La3134	Toxigenic	–	–	AV3134
DS	Nassarawa	Lafia	La3150	Atoxigenic	103.5	98.8	AV3150
DS	Nassarawa	Lafia	La3162	Atoxigenic	–	–	–
DS	Nassarawa	Lafia	La3193	Atoxigenic	122.2	98.6	–
DS	Nassarawa	Lafia	La3201	Toxigenic	–	–	AV3201
DS	Nassarawa	Lafia	La3224	Atoxigenic	282.4	96.7	AV3224
DS	Nassarawa	Lafia	La3228^c^	Toxigenic	8566.9	–	AV3228
DS	Nassarawa	Lafia	La3231	Toxigenic	–	–	AV3201
DS	Nassarawa	Lafia	La3279	Atoxigenic	17.1	99.8	AV3279
DS	Nassarawa	Lafia	La3303	Atoxigenic	25.9	99.7	AV3303
DS	Nassarawa	Lafia	La3304	Atoxigenic	44.1	99.5	AV3304
DS	Nassarawa	Lafia	La3305	Atoxigenic	–	–	AV3304
DS	Nassarawa	Lafia	La3306	Atoxigenic	22.9	99.7	AV3306
DS	Kogi	Lokoja	Lo4216	Toxigenic	–	–	AV4216
DS	Oyo	Ogbomosho	Og0104	Atoxigenic	–	–	AV3279
DS	Oyo	Ogbomosho	Og0106	Toxigenic	–	–	AV0106
DS	Oyo	Ogbomosho	Og0107	Toxigenic	–	–	AV0107
DS	Oyo	Ogbomosho	Og0165	Atoxigenic	51.2	99.4	AV0165
DS	Oyo	Ogbomosho	Og0173	Atoxigenic	499.3	94.2	AV0173
DS	Oyo	Ogbomosho	Og0205	Atoxigenic	177.4	97.9	AV0205
DS	Oyo	Ogbomosho	Og0216	Atoxigenic	138.0	98.4	–
DS	Oyo	Ogbomosho	Og0222	Atoxigenic	110.0	98.7	AV0222
DS	Oyo	Ogbomosho	Og0230	Atoxigenic	150.4	98.2	–
DS	Oyo	Ogbomosho	Og0425	Atoxigenic	–	–	AV0437
DS	Oyo	Ogbomosho	Og0437	Atoxigenic	333.1	96.1	AV0437
DS	Oyo	Ogbomosho	Og0440	Atoxigenic	–	–	AV0437
DS	Oyo	Ogbomosho	Og0452	Atoxigenic	297.7	96.5	AV0452
DS	Oyo	Ogbomosho	Og0479	Toxigenic	–	–	AV0479
LSD	–	–	–	–	179.0	–	–

a Adapted from our previous studies on distribution and toxigenicity of *Aspergillus* species in Nigeria (Atehnkeng *et al*., 2008a, 2008b).

b AEZ denotes agro‐ecological zone, where DS is derived savannah, NGS is northern Guinea savannah and SGS is southern Guinea savannah. FCT is the Federal Capital Territory State of Nigeria and LSD is Fisher's least significant difference at α = 0.05.

c Aflatoxin production is based amount produced when the isolate was co‐inoculated with the highly toxigenic isolate La3228 in aflatoxin‐free maize grain. RED denotes reduction (%) in aflatoxin production in co‐inoculation (*x*) of test isolate with toxin producing isolate La3228 based on toxin production by La3228 (*y*) as a reference and calculated as: RED (%) = 100 × [1 − (*x*/*y*)].

### Sclerotia size and number

**Figure 1:**
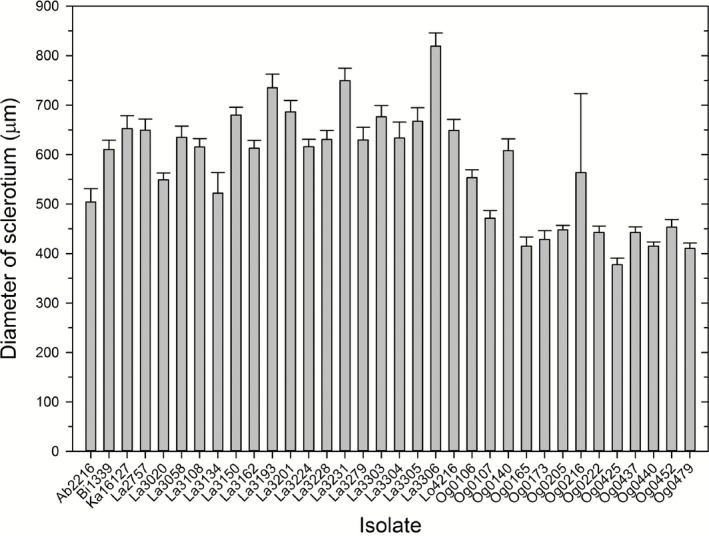
Size (i.e., diameter) of sclerotia produced by 34 isolates of *A* *spergillus flavus* collected from different agro‐ecological zones in Nigeria. Isolate Og0230 did not produce sclerotia in culture and has no corresponding value for size of sclerotia. For each bar, the whisker represents the standard error of the mean diameter of the sclerotia produced by the isolate.

All evaluated fungal isolates produced sclerotia except Og0230, for which no sclerotia were observed in culture on solid amended Czapek Dox medium. The diameter of sclerotia produced by fungal isolates was > 400 μm for all isolates that produced sclerotia except Og0425 that had a mean diameter of 377.5 μm (Fig. 1). Subsequent culturing of Og0425 on 5/2 medium did not result in sclerotia production, and thus, all isolates were identified to belong to the L‐strain morphotype of *A. flavus*. Highly significant differences in the number of sclerotia (*P* = 0.0084) and diameter of sclerotia (*P* = 0.0175) were observed between evaluated fungal isolates (Fig. 1). Isolate Og0173 obtained from Ogbomosho district in the DS zone produced the highest number of sclerotia, with a total of 121.8 sclerotia per square centimetre, whereas isolate Lo4216 from Lokoja district in the DS zone produced the fewest number of sclerotia, with a total of 16 sclerotia per square centimetre (data not shown). The largest sclerotia were produced by isolate La3306 from Lafia district in the DS zone, with a mean diameter of 819.0 μm, whereas the smallest sclerotia were produced by Og0425 from Ogbomosho district in the DS agro‐ecological zone, with a mean diameter of 377.5 μm (Fig. 1).

### Tester‐pair development for VCGs

Tester‐pairs, *cnx^−^* (defective in the molybdenum cofactor) and *niaD^−^* (defective in the structural gene for nitrate reductase) mutants were obtained for 31 isolates out of the 35 isolates that were initially selected. Fungal isolates for which testers were obtained generated spontaneous *nit^−^* mutants 5–15 days after inoculating solid Czapek–Dox–chlorate medium plates. Four isolates, La3162, La3193, Og0216 and Og0230, did not generate compatible tester‐pairs after more than 10 tester development efforts and were thus not assigned to any VCG (Table 1). Additional studies were not conducted to determine whether these four isolates were either members of a single VCG or whether they belonged to multiple VCGs.

A total of 25 VCGs were inferred from the 31 isolates for which tester‐pairs were obtained (Table 1). These VCGs were designated with an AV prefix denoting *Aspergillus* VCG followed by the number of the type isolate within the VCG, as new VCGs were discovered. Of the 25 identified VCGs, 20 consisted of a single isolate of *A. flavus*, whereas only 5 (AV0437, AV2216, AV3201, AV3279 and AV3304) were represented by either 2 or 3 more fungal isolates (Table 1). VCGs that were represented by more than one isolate were either toxigenic (e.g., AV2216 or AV3201) or atoxigenic (e.g., AV0437 or AV3279) and no identified VCG had both toxigenic and atoxigenic isolates.

Nine toxigenic VCGs were identified in this study, of which seven were represented by a single isolate and two were represented by two isolates. Of the 16 atoxigenic VCGs identified, 13 consisted of only 1 isolate, whereas 3 VCGs consisted of 2 or 3 fungal isolates (Table 2). Only one toxigenic VCG, AV2216, was found in two agro‐ecological zone, whereas most of the toxigenic or atoxigenic VCGs were found primarily in the DS zone (Table 2).

**Table 2:** Distribution of vegetative compatibility groups of *A* *spergillus flavus* within and between agro‐ecological zones and positive complementations with toxigenic and atoxigenic isolates in Nigeria

AEZ^a^	VCG^b^	Number of positive complementations^c^	Number of toxigenic positives^d^	Number of atoxigenic positives^d^	Designation of VCG
DS	AV0205	1	0	1	Atoxigenic
DS	AV3224	4	0	4	Atoxigenic
DS	AV3228	6	2	4	Toxigenic
DS	AV3201	13	3	10	Toxigenic
DS	AV0165	3	0	3	Atoxigenic
DS	AV0452	2	0	2	Atoxigenic
DS	AV3279	25	0	25	Atoxigenic
DS	AV0222	9	0	9	Atoxigenic
DS	AV0479	14	8	6	Toxigenic
DS	AV0437	9	0	9	Atoxigenic
DS	AV3306	1	0	1	Atoxigenic
DS	AV0106	6	2	4	Toxigenic
DS	AV3134	2	2	0	Toxigenic
DS	AV3108	1	0	1	Atoxigenic
DS	AV0107	18	9	9	Toxigenic
DS	AV3150	1	0	1	Atoxigenic
DS	AV3303	1	0	1	Atoxigenic
DS	AV3304	6	0	6	Atoxigenic
DS	AV0173	9	0	9	Atoxigenic
DS	AV4216	3	1	2	Toxigenic
SGS	AV3020	3	1	2	Toxigenic
SGS	AV3058	2	0	2	Atoxigenic
SGS	AV2757	1	0	1	Atoxigenic
SGS	AV2216	8	1	7	Toxigenic
NGS	AV16127	5	0	5	Atoxigenic
Total	–	153	29	124	–

a AEZ denotes agro‐ecological zone in which a VCG was found, where DS is derived savannah, SGS is southern Guinea savannah and NGS is northern Guinea savannah.

b VCG refers to vegetative compatibility group for each *A. flavus* isolates.

c Number of positive complementation by each VCG based on 832 *nit^−^* mutants.

d Number of corresponding positive complementations of each VCG with toxigenic or atoxigenic isolates.

### Inter‐location complementation

**Figure 2:**
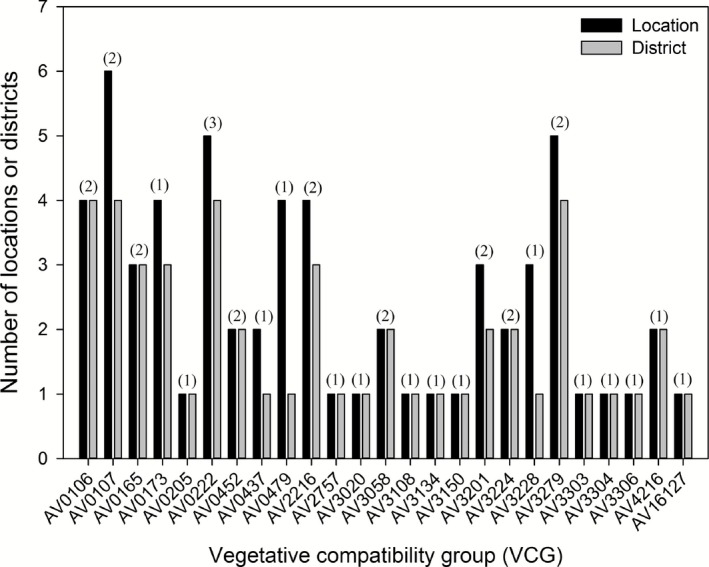
Distribution of *A* *spergillus flavus* vegetative compatibility groups (VCGs) across locations and districts in the three agro‐ecological zones in Nigeria. Numerals above a pair of bars for each VCG denotes the number (minimum = 1 and maximum = 3) of agro‐ecological zones where the VCG was present.

Out of 20,800 complementation tests (832 *nit^−^* mutants × 25 VCGs) performed in this study, 153 positive complementations were recorded, of which about 19% and 81% were accounted for by toxigenic and atoxigenic VCGs respectively (Table 2). A total of 62 positive complementations (40.5%) occurred between tester‐pairs and unknown *A. flavus* isolates from the same district. At other hierarchical levels, 49 positive complementations (32.0%) occurred between tester‐pairs and unknown isolates from different districts, whereas 38 (24.8%) positive complementations occurred between isolates in different agro‐ecological zones. Among atoxigenic VCGs, AV3279 was the mostly widely distributed with 25 positive complementations (16.3% of the total) (Table 2). AV3279 was also found in five locations and four districts and in two agro‐ecological zones, DS and southern Guinea savannah (SGS) zones (Fig. 2). The next most widely distributed atoxigenic VCGs were AV0173, AV0222, AV0437, AV3304 and AV16127, which had six to nine atoxigenic positive complementations (Fig. 2).

AV0107 was the most widely distributed toxigenic VCG with 18 positive complementations, out of which 9 were toxigenic positives (Table 2). AV0107 was present in six locations and four districts and was also present in both DS and SGS zones (Fig. 2). Ten VCGs (40%) were restricted to only one location, five VCGs (20%) were found in two locations, three (12%) in three locations, four (16%) in four locations, two (8%) in five locations, and one (4%) in six locations (Fig. 2). VCGs identified in this study were distributed in different proportions within the 11 surveyed districts. There were 13 VCGs (52%) that complemented with *nits^−^* from one district, five (20%) with *nits^−^* from two districts, three (12%) with *nits* in three districts, and four (16%) complemented with *nits^−^* in four districts. One VCG (4%), AV0222, originated from 3 agro‐ecological zones, 9 VCGs (36%) were found in 2 agro‐ecological zones, whereas 15 (60%) originated from 1 agro‐ecological zone (Fig. 2).

### Relative physical distances between members of VCGs

**Figure 3:**
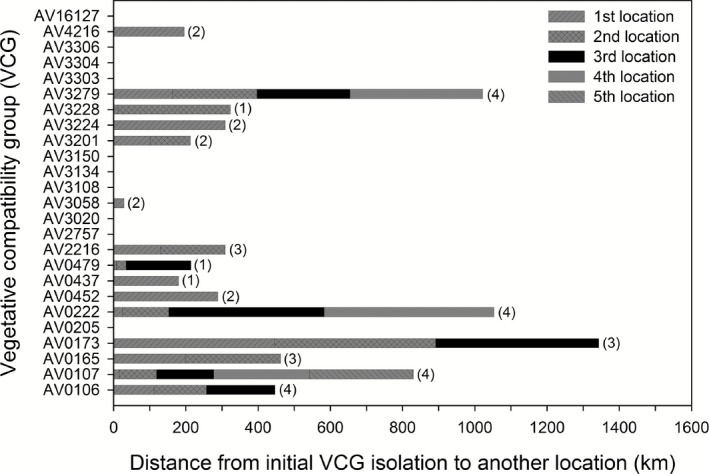
Distance map depicting the distance from the location where a vegetative compatibility group (VCG) was initially isolated to another location where a member of the same VCG was recovered. A VCG without a corresponding horizontal bar is a VCG not recovered beyond the original location where it was isolated. Numbers in parenthesis next to the horizontal bars are districts in which members of the VCG were recovered; VCGs without corresponding numbers had members recovered only in one district where the VCG was initially isolated.

Fifteen VCGs had *A. flavus* members that were recovered from multiple locations besides the original location where the same VCG was isolated, whereas 10 VCGs had no members recovered elsewhere (Fig. 3). Linear distances between members of the same VCG recovered in different locations ranged from 8 km for AV0479, to 481 km for AV0173 (Fig. 3). VCGs AV0173, AV0222 and AV3279 had members that were recovered from a total distance spanning 1,000–1,400 km. Ten VCGs (40%) had members restricted to their original point of isolation. One VCG (4%) had members distributed across less than 100 km, seven VCGs (28%) had members recovered between 100 and 200 km, two VCGs (8%) had members recovered between 200 and 300 km, three VCGs (12%) had members recovered between 300 and 400 km, and two VCGs had members (8%) distributed across > 400 km. The atoxigenic AV3279 was the most frequently detected VCG, with members found in other locations that were 368 km apart. The widely distributed toxigenic AV0107 was found in five locations spanning 288 km (Fig. 3).

### Genetic diversity of VCGs

The overall Shannon index of VCG diversity across the three agro‐ecological zones was 2.60 for VCGs identified in this study. The DS zone had the highest VCG diversity index (*H* = 2.61) compared with the SGS (*H* = 1.83) and northern Guinea savannah (NGS) (*H* = 0.94) agro‐ecological zones (Table 3). Given that the number of unique VCGs across the region was 25, the maximum possible level of richness, *H*_max_, was 3.22. Evenness of VCGs in the agro‐ecological zones was low and ranged from *E*_5_ = 0.63 in the SGS zone to *E*_5_ = 0.84 in the NGS zone (Table 3). The overall evenness of VCGs across the three agro‐ecological zones was *E*_5_ = 0.96. VCG richness based on rarefaction analysis was lowest in the NGS zone (E[*g*_9_] = 3.00) and highest in DS zone (E[*g*_9_] = 6.57).

**Table 3:** Diversity of vegetative compatibility groups in a population of *A* *spergillus flavus* collected from three agro‐ecological zones in Nigeria

VCG^a^	Agro‐ecological zone					
	DS^b^		SGS^b^		NGS^b^	
	*n* c	*H* d	*n* c	*H* d	*n* c	*H* d
AV0106	4	0.14	2	0.11	0	0.00
AV0107	14	0.30	4	0.18	0	0.00
AV0165	1	0.05	2	0.11	0	0.00
AV0173	9	0.24	0	0.00	0	0.00
AV0205	1	0.05	0	0.00	0	0.00
AV0222	6	0.19	2	0.11	1	0.24
AV0452	1	0.05	1	0.07	0	0.00
AV0437	9	0.24	0	0.00	0	0.00
AV0479	14	0.30	0	0.00	0	0.00
AV2216	1	0.05	7	0.25	0	0.00
AV2757	0	0.00	1	0.07	0	0.00
AV3020	0	0.00	3	0.15	0	0.00
AV3058	1	0.05	1	0.07	0	0.00
AV3108	1	0.05	0	0.00	0	0.00
AV3134	2	0.09	0	0.00	0	0.00
AV3150	1	0.05	0	0.00	0	0.00
AV3201	1	0.05	12	0.32	0	0.00
AV3224	1	0.01	0	0.00	3	0.37
AV3228	6	0.19	0	0.00	0	0.00
AV3279	1	0.05	24	0.37	0	0.00
AV3303	1	0.05	0	0.00	0	0.00
AV3304	6	0.19	0	0.00	0	0.00
AV3306	1	0.05	0	0.00	0	0.00
AV4216	3	0.12	0	0.00	0	0.00
AV16127	0	0.00	0	0.00	5	0.33
Total	85	–	59	–	9	–
*H* d	–	2.61	–	1.83	–	0.94
*E*_5_ e	–	0.74	–	0.63	–	0.84
*R*(*g*_9_)^f^	–	6.57	–	4.79	–	3.00
*VD* g	–	0.26	–	0.19	–	0.33

a VCG denotes vegetative compatibility to which each *A. flavus* isolate belong.

b DS is derived savannah, SGS is southern Guinea savannah, and NGS is northern Guinea savannah.

c Number of *A. flavus* isolates in each VCG group and agro‐ecological zone for a total of 85, 59 and 9 isolates in DS, SGS and NGS agro‐ecological zones respectively.

d *H* denotes Shannon index for genetic diversity in each VCG group and agro‐ecological zone.

e Evenness estimated as described in Grünwald and colleagues (2003) and is a measure of how VCG groups are distributed in each agro‐ecological zone.

f Richness expressed as expected number of VCGs estimated by rarefaction analysis for the smallest sample of isolates in the NGS zone (*n* = 9).

g VCG diversity based on number of unique VCGs and total number of isolates in each zone.

VCG diversity across the three agro‐ecological zones, expressed as the number of VCGs divided by the total number of fungal isolates in each VCG, was low to moderate, with VCG diversity higher in DS (*VD* = 0.26) and NGS (*VD* = 0.33) zones, but lower in the SGS (*VD* = 0.19) agro‐ecological zone. Overall, different indices indicated that the diversity of *A. flavus* VCGs was high in the DS zone, but low in the NGS and SGS agro‐ecological zones (Table 3).

## Discussion

This study represents the first documentation of the VCG structure of *A. flavus* and the diversity of toxigenic and atoxigenic VCGs of *A. flavus* associated with maize grain in major maize production areas in Nigeria. A major goal of this study was to generate quantitative information on the diversity of *A. flavus* VCGs and their distribution in the region, and integrate VCG diversity results with molecular characterization data to inform identification, selection and deployment of atoxigenic VCGs for biocontrol of toxigenic isolates to reduce aflatoxin contamination in maize. Our results indicate that the diversity of VCGs in *A. flavus* communities in Nigeria is high, and widely distributed indigenous atoxigenic VCGs of *A. flavus* present a unique opportunity to deploy potential biocontrol agents across different agro‐ecological zones to reduce aflatoxin contamination in maize.

Some of the widely distributed atoxigenic VCGs reported in the present study were previously identified for field testing (Atehnkeng *et al*., 2014) using data on efficacy in reducing aflatoxin under laboratory conditions (Atehnkeng *et al*., 2008b) and deletion patterns in the aflatoxin gene cluster (Donner *et al*., 2010). However, quantitative data on environmental distribution and genetic diversity of *A. flavus* VCGs and application of this information to facilitate selection and deployment of atoxigenic VCGs to mitigate aflatoxin contamination in maize was lacking prior to the present study. Knowledge of the environmental distribution and VCG diversity presented in this paper together with information on molecular characterization (Donner *et al*., 2009) and field efficacy (Atehnkeng *et al*., 2014) generated earlier have been used to select VCGs to constitute the multi‐strain biocontrol product Aflasafe™ for commercial use in Nigeria (Bandyopadhyay and Cotty, 2013; Grace *et al*., 2015). VCGs represent naturally occurring multilocus genotypes that are helpful to characterize genetic structure in fungal communities (Bayman and Cotty, 1993). Fungal isolates belonging to the same VCG are typically more closely related than isolates that belong to different VCGs, and there is no evidence to suggest that gene flow occurs between VCGs in nature (Grubisha and Cotty, 2010; 2015). Thus, VCG analysis is a useful tool to estimate genetic diversity and understand population dynamics and provides a means of identifying and characterizing diversity and distribution of *A. flavus* populations (Bayman and Cotty, 1991b; 1993; Barros *et al*., 2006). In the present study, we categorized locally adapted and endemic atoxigenic *A. flavus* VCGs to identify unique members within a given VCG that meet the criteria for selection as potential biocontrol agents. For safe application in the field, atoxigenic isolates of *A. flavus* selected as potential biological control agents should ideally be indigenous, genetically stable and must belong to a VCG that do not have toxigenic members (Cotty, 2006; Cotty *et al*., 2008; Mehl *et al*., 2012).

Diversity of VCGs across the region as estimated by Shannon's index of diversity (*H* = 2.60) was high, indicating that considerable diversity of *A. flavus* isolates is present in Nigeria. This high level of diversity is comparable with values reported for *A. flavus* in Italy where Shannon's indices of diversity ranged from 2.4 to 3.2 (Mauro *et al*., 2013). However, the overall diversity value reported in the present study is considerably higher than diversity values reported in Georgia, *H* = 0.69 (Papa, 1986), Argentina, *H* = 0.64 (Pildain *et al*., 2004) or Arizona, *H* = 0.54 (Bayman and Cotty, 1991b). Further, diversity was higher in the DS, compared with SGS and NGS agro‐ecological zones of Nigeria. As reported by Grünwald and colleagues (2003), genetic diversity depends on both the number of genotypes in a sample genetic (i.e., richness) and the distribution of genotypes in a population (i.e., evenness), with a higher level of genetic diversity being observed for a larger sample size. A similar trend was also observed in this study where the DS zone that had a larger sample size had a higher diversity compared with NGS zone that had a smaller sample size and a lower diversity. The SGS zone that had an intermediate sample size was associated with a corresponding intermediate level of diversity. In contrast, evenness was higher in NGS zone that had a lower sample size compared with DS zone, which had a larger sample size. The lower sample size in NGS was due to the limited maize production in this zone, which is warmer and drier and less suitable for maize production. A larger sample size not only increases genetic richness, but also reduces evenness by increasing the probability of sampling several isolates belonging to the same genotype. Thus, the higher *H* value in DS zone was probably affected by the larger sample size, whereas the evenness values indicate a more uniform distribution of *A. flavus* genotypes in NGS zone compared with DS or SGS agro‐ecological zone.

The genetic richness value of *H* = 2.60 across the region represents 80.7% of the maximum possible value (*H*_max_), which strongly indicates a higher degree of diversity of *A. flavus* population in Nigeria. In addition, the high overall evenness of isolates across the region (*H* = 0.96), which is indicative of the presence of very few dominant VCGs in the population, also supports our observation on the high degree of diversity for *A. flavus* population in this study. A similarly high degree of diversity was also reported in populations of *A. flavus* in Louisiana (Sweany *et al*., 2011). Although cryptic sexual reproduction has been reported in *A. flavus* (Moore *et al*., 2013), population studies have indicated that the fungus propagates in nature through asexual production of conidia that are readily dispersed aerially over a large area (Grubisha and Cotty, 2010). Thus, the high degree of diversity reported in this study may be explained in part by the introduction of previously isolated and genetically distinct individuals and a high rate of mutation events over very long periods as has been reported for rust pathogens (Keiper *et al*., 2006).

In this study, all isolates were classified as belonging to the L‐strain morphotype of *A. flavus*. However, the analysis of variance showed significant differences in the number and size of sclerotia within and between VCGs. These observations are similar to those of Novas and Cabral (2002) who also found differences in the number and size of sclerotia between isolates belonging to the same VCG. Thus, although it is clear that distinct VCGs are clonal lineages, individuals within VCGs are likely to continue to accumulate genetic mutations (Grubisha and Cotty, 2010). These accumulated mutations can give rise to varying phenotypes, such as differences in number and size of sclerotia, and differences in toxin production within a VCG. Indeed there is significant evidence for the potential for rapid divergence among individuals within clonal lineages of other fungi and oomycetes, resulting in changes in virulence and host range (Goss *et al*., 2009).

Sixteen atoxigenic VCGs with varying levels of positive complementations with atoxigenic isolates ranging from 1 to 25 were identified in this study. Membership in a VCG with only atoxigenic members is one of the critical criteria for selecting isolates of *A. flavus* that are intended for use as biocontrol agents. Although concerns over potential recombination events between endemic atoxigenic biocontrol and endemic toxigenic isolates of *A. flavus* have been expressed (Geiser *et al*., 1998), population genetic analyses have failed to detect gene flow between different VCGs (Grubisha and Cotty, 2010). Vegetative incompatibility barriers suggest that the exchange of genetic material is more likely to occur between isolates within a VCG than between isolates in different VCGs. Thus, atoxigenic isolates that belong to VCGs with toxigenic members should not be selected as potential biocontrol isolates. In this study, VCGs such AV0222, AV0437, AV3279 and AV16127, had a high number of positive complementations for which none were with toxigenic isolates. Thus, atoxigenic isolates such as La3279, Og0222, Og0437 or Ka16127 that belong to these specific VCGs are potential candidate isolates for use in biocontrol of aflatoxin in maize in the region.

Deployment of an atoxigenic biocontrol isolate that belongs to a widely distributed atoxigenic VCG is one of the key elements in the sustainable use of biocontrol. In this study, we identified atoxigenic VCGs that had members found in several locations in different agro‐ecological zones. For example, *A. flavus* isolates that were members of AV3279 and AV0222 were found in two and three agro‐ecological zones, across a linear distance of 368 km and 470 km respectively. This observation suggests that atoxigenic members of AV3279 and AV0222 may be adapted to a wide geographical range and could be used for region‐wide biocontrol of aflatoxin in maize in Nigeria. Atoxigenic isolates that are naturally adapted and belonging to a widespread VCG are likely to be better competitors when applied in the field as biopesticides. In this study, mutants of four isolates were unable to complement with any other mutants derived from the same isolate. This failure of complementation could be due to heterokaryon self‐incompatibility, in which fungi are unable to form stable heterokaryon with themselves as reported in *Fusarium* species and other imperfect fungi (Brooker *et al*., 1991; Clark *et al*., 1995; Harveson and Rush, 1997). Such isolates usually complicate population genetic analysis because it is difficult to assign them to any VCG. In this study, isolates La3162, La3139, Og0216 and Og0230 had *nit^−^* mutants that did not complement with other mutants derived from the same isolates and were thus not assigned to a VCG. An isolate that is unable to complement with mutants derived from the same isolate is not potentially useful for biocontrol because of the difficulty of tracking them in the field.

Atoxigenic VCGs such as AV0222, AV3279, AV3304 and AV16127 were widely distributed across the region. Thus, *A. flavus* isolates belonging to these VCGs could further be characterized for use as potential biocontrol agents for the suppression of aflatoxin in maize. To understand the nature of atoxigenicity for members within these atoxigenic VCGs, we compared the aflatoxin biosynthesis gene cluster (Donner *et al*., 2010) for the 16 atoxigenic VCGs with that of two toxigenic isolates from Nigeria and an isolate belonging to the VCG group of AF36, a commercial biocontrol product in the US. In that study (Donner *et al*., 2010), sequences from 14 genes from the aflatoxin gene cluster were generated to assess nucleotide polymorphism and 8 additional cluster genes were polymerase chain reaction‐amplified to determine the presence or absence of the genes. Further, relationships among the examined isolates were assessed with phylogenetic analyses that included two additional protein‐coding loci outside the aflatoxin gene cluster, *pecA* and *taka amylase*. Isolates of *A. flavus* in AV0222 had deletion of the entire aflatoxin gene cluster, whereas isolates in AV0452 and AV0173 had large deletions that included the 5′end but with remnants of the *norB‐cypA* region (Donner *et al*., 2010). Isolates in AV0165 contained large lesions extending to *ordB* and *hypA*, at the distal end of the aflatoxin gene cluster. As was the case with the AF36 isolate, fungal isolates in AV3279, AV3304 and AV16127 also lacked deletions in portions of the aflatoxin biosynthesis gene cluster examined. Based on VCG diversity and molecular data, atoxigenic *A. flavus* isolates belonging to the widely distributed VCGs such as AV0173, AV0222, AV3279 and AV3304 can be selected as biocontrol agents for region‐wide deployment in Nigeria. Atoxigenic isolates belonging to AV0222, AV3279, AV3304 and AV16127 have previously been selected and constitute the active ingredients in Aflasafe™, a recently commercialized product for biocontrol of aflatoxin in Nigeria (Bandyopadhyay and Cotty, 2013; Schmidt, 2013). The high diversity of VCGs in *A. flavus* communities in this study suggests that a single atoxigenic isolate is likely to be less effective in reducing aflatoxin contamination in maize in different regions in Nigeria. An alternative strategy would be to use a mixture of atoxigenic isolates belonging to widely distributed VCGs such as AV0173, AV0222, AV3279 and AV3304. The use of a mixture of atoxigenic isolates that belong to widely distributed VCGs can overcome challenges that plague biocontrol such as specificity of efficacy in limited agro‐ecological zones and thus result in increased efficacy across complex cropping systems (Probst *et al*., 2011).

The present study established that *A. flavus* communities across three agro‐ecological zones in Nigeria are genetically very diverse. Genetic diversity in *A. flavus* can be generated by a variety of mechanisms and has important implications on the selection and deployment of atoxigenic isolates in the biocontrol of aflatoxin. Sexual recombination can generate diversity in *A. flavus* (Moore *et al*., 2013), but there is no direct evidence to indicate that it occurs under natural conditions in the field (Grubisha and Cotty, 2010). We identified indigenous atoxigenic isolates that are widely distributed and adapted to local farming systems, target crops and environments; and these atoxigenic isolates can be useful in aflatoxin biocontrol programmes. These atoxigenic isolates were members of specific atoxigenic VCGs that were widespread across DS, SGS and NGS agro‐ecological zones. Deployment of members of indigenous atoxigenic VCGs that are widely distributed and genetically similar to the local soil populations of *Aspergillus* communities and thus adapted to the target region will increase the efficacy of biocontrol (Mehl *et al*., 2012; Lewis *et al*., 2013) and in the process result in an ecologically sustainable biocontrol of aflatoxin contamination in maize. Recent studies conducted under laboratory conditions have shown that stress biology‐related factors such as solutes, temperature, humidity and water activity can impact growth and aflatoxin production in *Aspergillus* species (de Lima Alves *et al*., 2015; Medina *et al*., 2015; Stevenson *et al*., 2015). An improved understanding of the impact of these environmental factors on the stress biology of *A. flavus* under field conditions could also be useful in improving the efficacy of biocontrol of toxigenic isolates (Cray *et al*., 2015).

## Experimental procedures

### Fungal isolates and quantification of aflatoxin production

A total of 35 isolates of *A. flavus* were obtained from maize grains collected from farmers' stores in SGS, DS and NGS agro‐ecological zones of Nigeria in 2008 and 2009 and subjected to detailed analyses (Table 1). Temperatures increase and precipitation decreases with increasing latitude with the DS being the southernmost, followed by the SGS and the NGS in the north. These 35 isolates were selected (at least one from each agro‐ecological zone) from 4,237 *Aspergillus* section *Flavi* isolates collected from maize samples from 52 locations in 11 administrative districts across the three agro‐ecological zones (Atehnkeng *et al*., 2008b). The 35 selected isolates that were atoxigenic in the initial assessment of aflatoxin‐producing ability were retested by inoculating aflatoxin‐free maize grain and analyzing the extracts for aflatoxin content 7 days after inoculation as described by Probst and colleagues (2014). Confirmed atoxigenic isolates were tested for their inability to produce aflatoxin by running the aflatoxin production assay at least three times for each isolate. Selected atoxigenic isolates were further co‐inoculated with a highly toxigenic *A. flavus* L‐strain isolate La3228, previously obtained from maize in Nigeria, to determine their relative competitiveness in aflatoxin reduction in maize grain. Briefly, co‐inoculations were conducted in the laboratory by inoculating 30 sterilized maize kernels in a vial with 10 μl of inoculum containing 1 × 10^6^ conidia ml^−1^ each of the selected atoxigenic isolate and La3228. Isolate La3228 is highly toxigenic and produces high quantities of aflatoxin in the order of 24,000 ppb (Atehnkeng *et al*., 2008a). Vials were then shaken in a Vortex mixer for 30 s to ensure complete and uniform coating of kernels with the inoculum and then incubated at 31°C for 7 days. Thereafter, vials were thereafter oven dried at 45°C for 1 day to halt any fungal activity, and kernels were prepared for aflatoxin extraction and analysis. Dry extracts from test kernels and aflatoxin standards were separated on thin‐layer chromatography (TLC) plates (silica gel 60, 250 μm) using the diethyl ether–methanol–water (96:3:1) solvent (Cotty, 1997). The level of aflatoxin in the test samples was quantified as described by Atehnkeng and colleagues (2008a) using a scanning densitometer, CAMAG TLC Scanner 3 with WinCATS 1.4.2 software (Camag AG, Muttenz, Switzerland) with 0.1 ng g^−1^ detection limit.

### Size and number of sclerotia

*Aspergillus flavus* isolates can morphologically be classified into either an S‐ or L‐strain depending on the size and number of sclerotia and production of sclerotia on 5/2 medium. Typically, S‐strain isolates produce smaller sclerotia with a mean diameter of < 400 μm on Czapek–Dox medium with 3% NaN0_3_, and they also produce sclerotia on 5/2, whereas L‐strains produce larger sclerotia on amended Czapek–Dox medium with a mean diameter of > 400 μm, but do not produce sclerotia on 5/2 medium or Czapek–Dox medium lacking NaN0_3_ (Cotty, 1989). To determine the production of sclerotia, Petri plates (dia. 9 cm) containing Czapek–Dox medium, were inoculated with spore suspension adjusted to 1 × 10^6^ conidia ml^−1^ of each of the 35 *A. flavus* isolates (i.e., treatments). Sclerotia were obtained by modifying the method described by Barros and colleagues (2006) by pouring 10 ml of water containing Tween 20 (0.01%) per plate, washing off the spores and rinsing the surface of culture plates with tap water 5–7 days after inoculation. Cultures were incubated in the darkness at 31°C for 14 days (Novas and Cabral, 2002; Pildain *et al*., 2004). Inoculated plates were laid out in the incubator in a complete randomized design and with three replications. Each isolate was then scored for the presence or absence of sclerotia 2 weeks after incubation.

Counts of the number of sclerotia were recorded using a grid method (Novas and Cabral, 2002; Pildain *et al*., 2004). Briefly, lines were marked at the bottom of 9 cm Petri plate to form 1 cm^2^ grids and the number of sclerotia was obtained by counting sclerotia in three arbitrarily selected grids for each replication. The spheroid sclerotia were then spread out on a microscope slide and the diameter of 20 randomly selected sclerotia per replicate was recorded under a calibrated microscope (Leitz Laborlux S, Germany) at a 10× magnification.

### Tester‐pair development for VCGs

To determine the diversity and distribution of VCGs across different agro‐ecological zones, 35 isolates of *A. flavus* described above were used for VCG tester‐pair development. Nitrate non‐utilizing (*nit^−^*) mutants were generated using a modified method of Bayman and Cotty (1991a). Briefly, fungal isolates were grown on a selective medium [Czapek–Dox broth (Difco)] containing 25 g l^−1^ potassium chlorate, 50 mg l^−1^ rose Bengal and 20 g l^−1^ agar with pH adjusted to 7. The selective medium was inoculated with a conidial suspension of *A. flavus* in a well at the centre of a 9 cm Petri plate. Cultures were incubated at 31°C, and margins of colonies with restricted growth were periodically examined for fast‐growing sectors containing sparse mycelium. Hyphal tips from sectors arising from different colonies were transferred to Petri plates containing Czapek–Dox broth with 15 g l^−1^ potassium chlorate and 20 g l^−1^ agar with pH adjusted to 7 to stabilize the mutants and confirm their inability to utilize nitrate. The *nit^−^* mutant phenotypes, *niaD^−^* (defective in the structural gene for nitrate reductase), *nirA^−^* (defective in the nitrate reductase) and *cnx^−^* (defective in the molybdenum cofactor) were determined by growing the mutants on a medium with nitrite, hypoxanthine or ammonium as sources of nitrogen as previously described (Cove, 1976; Bayman and Cotty, 1991a). A complementary pair of nitrate non‐utilizing auxotrophs composed of either a *niaD^−^* and a *cnx^−^* or a *niaD^−^* and a *nirA^−^* mutant was obtained for each isolate, and complementary pairings were first conducted to establish self‐compatibility (Bayman and Cotty, 1991a). Complementary pairs of mutants from an isolate were used as tester‐pairs and complementation with one or both of the tester mutants of a VCG‐defined membership in that VCG.

### Frequencies of VCGs within *A* *. flavus* populations

To determine the distribution of VCGs in the areas surveyed, *nit^−^*mutants were developed from 16 isolates in all of the 52 locations, which resulted in a total of 832 mutants. All the *nit^−^* mutants were used to perform complementation test with each VCG tester‐pair. Complementation tests were conducted by placing 10 μl of a spore suspension of each member of a VCG‐defining tester‐pair and a *nit^−^* mutant (unknown phenotype) of 1 of the 832 isolates in 3 mm wells cut into complementation medium. The wells were arranged approximately 15 mm apart in a triangular pattern so that each tester may react with both the *nit^−^* mutant and the other tester. The Petri plates were incubated at 31°C from 7 to 10 days. Compatibility was identified by a line of prototrophic growth, where the mycelia interacted. This interaction was frequently associated with formation of sclerotia.

### Inter‐location complementation

Distance between a VCG that was found in other locations apart from the location where the VCG was initially isolated was estimated to determine the distribution of the VCG within the surveyed locations. These distances were estimated using coordinates of the sampled locations obtained from a hand‐held Global Positioning System device (eTrex GPS, Garmin Corporation, Olathe, KS, USA).

### Statistical analyses

Aflatoxin concentration from experiments involving co‐inoculation of atoxigenic isolates and the toxigenic isolate La3228, and number of sclerotia and diameter of sclerotia for each VCG, were subjected to statistical analysis using the MIXED procedure of sas (version 9.2; Cary, NC, USA). Means for aflatoxin concentration, number of sclerotia and diameter of sclerotia were separated using Fisher's protected least significant difference test to determine differences between fungal isolates and VCGs. Linear distances between the original location where a VCG was isolated and other locations where the same VCG was found were used to indicate the extent to which a given VCG was distributed within the surveyed region. Diversity of VCGs in the three agro‐ecological zones was estimated using different approaches. First, VCG diversity was estimated based on Shannon's index (Shannon and Weaver, 1949) using the formula: *H* = [−Σ(*n_i_*/*N*) × ln(*n_i_*/*N*)], where *n_i_* is the number of isolates in the *i*th VCG cluster, and *N* is the total number of isolates, which was 85, 59 and 9 in DS, SGS, and NGS agro‐ecological zones respectively. The maximal value for Shannon's index (*H*_max_) was estimated as *H*_max_ = ln(*g*), where *g* denotes the number of unique VCGs (Grünwald *et al*., 2003). Two other diversity indices were also calculated; genetic evenness (*E_5_*) was calculated as: *E*_5_ = (*G* − 1)/(*N*_1_ − 1), where *G* = 1/[Σ(*n_i_*/*N*)^2^] and *N*_1_ = *e^H^* (Grünwald *et al*., 2003). Given that the total number of isolates in the three agro‐ecological zones was different, rarefaction analysis was conducted to compare VCG richness across zones. In this case, VCG richness was expressed as the expected number of VCGs, i.e., *E* = [*g*_9_], in a random sample of 9, the smallest sample of isolates in the NGS zone. Rarefaction analysis was conducted in R using the package *poppr* (Kamvar *et al*., 2014), which is specifically designed for analysis of populations that are clonal, admixed and/or sexual. VCG diversity was also estimated as the number of VCGs divided by the number of isolates (Mauro *et al*., 2013), with values ranging between 0 and 1, where a value of 1 indicates maximum diversity, whereby each isolate represents a distinct VCG.

## Conflict of interest

None declared.
